# Prevalence of occupational infectious diseases among primary oral health care teams and prevention measures

**DOI:** 10.47626/1679-4435-2020-546

**Published:** 2020-12-11

**Authors:** Sheila Cristina Vargas, Caio Fernando de Oliveira, Jane Dagmar Pollo Renner, Suzane Beatriz Frantz Krug, Lia Possuelo

**Affiliations:** 1 Pós-Graduação em Promoção da Saúde, Universidade de Santa Cruz do Sul - Santa Cruz do Sul (RS), Brazil

**Keywords:** communicable diseases, personal protective equipment, post-exposure prophylaxis

## Abstract

**Background:**

The dental surgeon and dental health assistant experience risk situations and must be prepared for the prevention of diseases and accidents at work.

**Objective:**

Evaluating the prevalence of occupational infectious diseases and prevention measures used by the DS and dental health assistants.

**Methods:**

Cross-sectional study carried out with professionals working at the Northwestern region of Rio Grande do Sul. We carried out an interview using a structured questionnaire containing variables related to sociodemographic factors and prevention of infectious diseases. Subjects were submitted to blood collection for serological test for infectious diseases.

**Results:**

The use of goggles and cap was higher among the dental health assistant. The dental surgeon reported frequent use of mask: 45.5% replaced the used mask at each new patient. All participants reported using gloves and changing them for each new patient. 78.3% of workers have attended patients with infectious diseases. Among professionals evaluated, dental surgeons reported the highest number of accidents with needle stick instruments (59.1%). Post-exposure prophylaxis procedures are known by 68.2% of dental surgeon and 62.5% of dental health assistant. We did not find positive results for hepatitis B, hepatitis C and human immunodeficiency virus (HIV).

**Conclusions:**

We did not found cases of occupational infectious disease. Dental professionals adopt some preventive actions, but a few professionals, even knowing about the risks they will be exposed during work, do not use some protective equipment.

## INTRODUCTION

Occupational diseases represent a work-related health problem. Health care professionals such as dental surgeons (DS) and dental assistants (DA) face risk situations daily and must be well informed about the prevention of diseases and occupational risks. Biological (bacteria, fungi, parasites), physical (noise, vibration, luminosity), chemical (dust, mercury handling, silver fillings), and ergonomic (posture, repetitiveness, excess effort) risks are present in the work environment of oral health care professionals.^1^

The first report of an accidental occupational contamination of health care professionals in Brazil is from 1984.^2^ Since then and despite the safety measures emphasized by the Brazilian Ministry of Health, such as the correct use of personal protective equipment (PPE), the number of professionals who have contracted diseases due to the exposure to occupational hazards has gradually increased.^3^ The adequate use of PPE indicates whether the professionals are aware of work-related risks and of how these utensils protect them from infectious diseases (among other hazards) and prevent cross-infections. Knowledge on infectious disease prevention in dentistry is crucial for DS and DA in order to prevent an increase in the prevalence of infectious diseases among both professionals and patients.^4^^,^^5^ The use of PPE provides DS, DA, and patients with a more reliable and safe care, reducing the possibility of infectious microorganism transmission as well as risks of cross-infection and occupational diseases.^6^

Oral health care professionals face direct contact with invasive microorganisms and this susceptibility contributes to increased infections with hepatitis B, hepatitis C, tuberculosis, and the human immunodeficiency virus (HIV).^7^ The biological risk in their work environment is related to the use of contaminated materials and to the direct contact with patients that carry infectious agents.^8^^,^^9^ The degree of exposure to these diseases depends on the correct handling of needles and sharp instruments and the risk of contamination with organic fluids,^10^ which can be increased by accidental close contact with a sick or infected individual. In addition to the accidental exposure to biological material, DS might also be exposed during the clinical procedure through aerosols or splatter.^11^

Postexposure prophylaxis (PEP) is a crucial element of work-related infectious disease prevention programs and includes procedures that should be performed after the exposure to microrganisms.^5^ These procedures include immediate washing of the exposed surface, determination of the risk associated with the exposure, and evaluation of the source patient for the following characteristics: presence of acquired immunodeficiency syndrome (AIDS) or hepatitis B or C infections, hepatitis B vaccination, intake of antiretroviral drugs, and evaluation and follow-up of hepatitis C.^12^

The aim of this study was to evaluate the prevalence of occupational infectious diseases and the knowledge and practice of prevention measures by DS and DA working within a regional health care coordinating body in the northwestern region of the state of Rio Grande do Sul, Brazil.

## METHODS

### STUDY DESIGN

This is a cross-sectional study performed at the 9th Regional Health Care Coordination Center of the Rio Grande do Sul state (*9ª* Coordenadoria Regional de Saúde/Rio Grande do Sul [9th CRS/RS]). The 9th CRS/RS is located in the state’s northwestern region, consisting of 13 municipalities with an overall population of 152,070 inhabitants. Its public primary health care network comprises 22 Family Health Strategy (FHS) units and 16 Primary Health Care (PHC) units, with 38 oral health teams employing 59 DS and 36 DA both male and female. All professionals who signed the free and informed consent form were included in the study. Professionals who were on vacation at the time of the researcher’s visit were excluded. After applying the inclusion and exclusion criteria, our sample consisted of 22 DS and 24 DA, totaling 46 participants.

### DATA COLLECTION

Data were collected through a structured questionnaire containing closed questions regarding the participant’s knowledge on the exposure to infectious diseases and the use of PPE. Interview appointments were performed with prior scheduling according to the routine of the oral health care team. The questionnaire evaluated demographic and socioeconomic variables, as well as variables related to occupational risk factors in the diagnosis and prevention of infectious diseases.

Levels of schooling were divided into two categories: 11 years of study (high school) or more than 11 years of study (technical school, incomplete college/university, complete college/university, and graduate education). The variables related to occupational risk factors were specialty, use of needles and sharp instruments, and use of PPE. Variables related to the diagnosis of infectious diseases were: tuberculin test for assessing latent tuberculosis (self-reported), HIV, hepatitis B core (HBc) antibody test, hepatitis B surface antigen (HBsAg) test, hepatitis B immunization test (anti-HBs), and hepatitis C virus (HCV) test. Finally, the variables used to evaluate the measures of prevention of infectious diseases were use of long-sleeved coat, cap, mask, goggles, and gloves; hand washing; environmental disinfection; sterility testing; and periodic autoclave maintenance. The vaccination status was assessed through the interview.

The economic classification of the assessed population used the criteria published by the Brazilian Association of Research Companies (Associação Brasileira de Empresas de Pesquisa [ABEP]); these are based on a score that considers the possession of consumer goods (television, refrigerator, radio, automobile, washing machine, DVD, freezer) and the level of schooling of the head of the family. Individuals were classified as belonging to the A, B, C, D, or E socioeconomic classes, with class A representing the highest socioeconomic class, and E the lowest class.

### LABORATORY ANALYSES

After the interview, DS and DA were invited to undergo blood collection via venipuncture of the brachial vein. Out of the 22 DS and 24 DA, 16 (73%) and 19 (79%), respectively, accepted the procedure. The blood samples were sent to the Genetics and Biotechnology Laboratory at the University of Santa Cruz do Sul (UNISC), where commercial immunochromatographic tests (Allere, Matsudo, Japan) were performed to detect HIV, HCV, HBsAg; anti-HBc and anti-HBs antibodies were determined using commercial immunoenzymatic assays and the Access 2 automated system (Beckman Coulter, Brea, USA). The technical procedures were performed as described by the manufacturers.

### ETHICAL CONSIDERATIONS

The study was approved in 2016 by the local ethics committee under protocol number 1824534 in accordance with resolution 466/2012 of the Brazilian National Health Council. The study participants expressed their consent by signing a free and informed consent form.

### STATISTICAL ANALYSES

Data analysis was performed through descriptive statistics of the variables and estimation of dispersion measures (mean, frequency, and standard deviation [SD]). Univariate analyses were performed for categorical variables using the chi-square test when comparing different groups using the SPSS 20.0 software. All statistical tests were considered significant when p < 0.05.

## RESULTS

The 22 DS and 24 DA included in this study represented 37.3% and 66.7%, respectively, of all professionals of each specialty in the assessed region. The sociodemographic characteristics of the study participants are shown in Table 1. Regarding the levels of schooling, 54.5% of the DS had graduate education and 50% of the DA had completed technical school or undergraduate studies.

**Table 1:** Sociodemographic and occupational characteristics of primary oral health care teams in Brazil.

Variable	DA n (%)	DS n (%)	Total N (%)	p-value*
Sex				
Male	0 (0.0)	11 (50.0)	11 (24.0)	0.43
Female	24 (100.0)	11 (50.0)	35 (76.0)	
Age range (years)				
20-30	10 (41.6)	9 (41.0)	19 (41.3)	0.53
31-39	6 (25.0)	6 (27.2)	12 (26.1)	
40-49	4 (16.7)	6 (27.2)	10 (21.7)	
50+	4 (16.7)	1 (4.6)	5 (10.9)	
Ethnicity				
White	20 (83.3)	20 (90.9)	40 (87.0)	0.66
Non-White	4 (16.7)	2 (9.1)	6 (13.0)	
Marital status				
With partner^†^	13 (54.2)	11 (50.0)	24 (52.1)	0.90
Without partner^‡^	11 (45.8)	11 (50.0)	22 (47.8)	
Schooling (years)				
Up to 11	12 (50.0)	0 (0.0)	12 (26.0)	0.48
More than 11	12 (50.0)	22 (100.0)	34 (74.0)	
Years worked				
Less than 5	12 (50.0)	13 (59.1)	25 (54.4)	0.92
More than 5	12 (50.0)	9 (40.9)	21 (45.6)	
Workload (hours/week)				
20	0 (0.0)	3 (13.6)	3 (6.5)	0.06
40	24 (100.0)	19 (86.4)	43 (93.5)	

DA: dental assistant; DS: dental surgeon.

* Chi-square test;

† married and stable union;

‡ single, divorced, or widowed.

Table 2 shows the professionals’ answers regarding the use of PPE. All participants of this study reported using gloves and exchanging them between patients. All interviewees also wore long-sleeved coats.

**Table 2:** Use of personal protective equipment (PPE) by primary oral health care teams in Brazil.

PPE	DA n (%)	DS n (%)	Total N (%)	p-value*
Goggles				
Always	13 (54.2)	10(45.5)	23 (50.0)	0.83
Sometimes	7 (29.2)	8 (36.4)	15 (32.3)	
Never	4 (16.7)	4 (18.2)	8 (17.4)	
Cap				
Always	18 (75.0)	14 (63.6)	32 (69.3)	0.66
Sometimes	4 (16.7)	6 (27.3)	10 (21.7)	
Never	2 (8.3)	2 (9.1)	4 (8.7)	
Mask				
Always	15 (62.5)	22 (100.0)	37 (80.4)	0.006
Sometimes	4 (16.7)	0 (0.0)	4(8.7)	
Never	5 (20.8)	0 (0.0)	5 (10.9)	
Mask change between patients				
Yes	7 (29.2)	10 (45.5)	17 (37.5)	0.25
No	17 (70.8)	12 (54.5)	29 (63.0)	

DA: dental assistant; DS: dental surgeon.

* Chi-square test.

Table 3 illustrates aspects of the occupational exposure of participants; despite a high prevalence of patients with infectious diseases, 30.1% of the interviewed professionals were unaware of the necessary prevention measures in the oral health care of patients with tuberculosis. When questioned about these measures, one (2.4%) professional stated that the correct conduct was to keep away from the patient, while 13 (37%) and 10 (28.3%) reported the need for complete PPE and a mask, respectively.

**Table 3:** Occupational exposure of primary oral health care teams in Brazil.

	DA n (%)	DS n (%)	Total N (%)	p-value*
Is aware of prevention measures for the oral health care of a patient with tuberculosis	18 (75.0)	14 (63.6)	32 (69.9)	0.40
Has already treated a patient with tuberculosis	6 (25.0)	10 (45.5)	16 (34.8)	0.14
Has already treated a patient with HIV	18 (75.0)	21 (95.5)	39 (84.8)	0.05
Has already treated a patient with hepatitis B	12 (50.0)	15 (68.2)	27 (58.7)	0.21
Has already treated a patient with hepatitis C	11 (45.8)	13 (59.1)	24 (52.2)	0.36

DA: dental assistant; DS: dental surgeon; HIV: human immunodeficiency virus.

* Chi-square test.

Table 4 illustrates the professionals’ history of periodic examinations, vaccination, and immunization according to the collected data. All DS and all but one DA had been vaccinated against hepatitis B. No positive results were observed on HBsAg, HBc, HCV, and HIV tests.

**Table 4:** Periodic examinations, vaccination, and immunization of primary oral health care teams in Brazil.

	DA n (%)	DS n (%)	Total N (%)	p-value*
Undergoes periodic HIV testing	17 (70.8)	10 (45.5)	27 (58.7)	0.08
Undergoes periodic testing for				
Hepatitis B	12 (26.1)	10 (45.5)	22 (47.8)	0.54
Hepatitis C	12 (26.1)	9 (19.6)	21 (45.7)	
Undergoes periodic testing for tuberculosis (tuberculin skin test)	12 (50.0)	9 (40.9)	21 (45.7)	0.53
Has an updated vaccination schedule	21 (87.5)	14 (30.4)	35 (76.1)	0.05
Hepatitis B vaccination				
Not vaccinated	1 (2.4)	0 (0.0)	1 (3.3)	0.54
Incomplete vaccination schedule	2 (8.3)	3 (6.5)	5 (10.9)	
Complete vaccination schedule	21 (87.5)	19 (86.4)	40 (87.0)	
Is anti-HBs reactive	17 (89.4)	9 (56.3)	26 (74.3)	0.25
Has a BCG vaccination scar	23 (95.8)	19 (86.4)	42 (91.3)	0.25

anti-HBs: hepatitis B immunization test; BCG: bacille Calmette-Guérin; DA: dental assistant; DS: dental surgeon; HIV: human immunodeficiency virus.

* Chi-square test.

Although DS presented the highest number of needlestick injuries, DA showed a higher frequency of accidents with visible blood (Table 5). When interviewed about PEP, 66.6% of DS and 80% of DA correctly answered some of the procedures contained in the protocol. The correctly reported procedures were accident notification, rapid HIV test, start of antiretroviral medication, and referral to specialized care.

**Table 5:** Characteristics and frequency of accidents with sharp instruments reported by primary oral health care teams in Brazil.

	DA n (%)	DS n (%)	Total N (%)	p-value*
Accidents with sharp instruments	5 (20.8)	13 (59.1)	18 (39.1)	0.08
Accidents with visible blood	2 (8.3)	1 (4.5)	3 (12.8)	0.06
Knows postexposure prophylaxis procedures	15 (62.5)	15 (68.2)	30 (65.2)	0.68
Reported postperforation procedures				
Notification	4 (26.7)	2 (13.3)	6 (20.0)	0.40
Rapid HIV test	12 (80.0)	11 (73.3)	23 (76.6)	
Sanitizing with water	7 (46.7)	8 (53.3)	15 (50.0)	
Sanitizing with 70% alcohol	1 (6.7)	1 (6.7)	2 (6.7)	
Applying pressure to the lesion	1 (6.7)	1 (6.7)	2 (6.7)	
Start medication	4 (26.7)	5 (33.3)	9 (30.0)	
Refer to SAS	8 (53.3)	7 (46.7)	15 (50.0)	
Refer to hospital	0 (0.0)	1 (6.7)	1 (3.3)	

DA: dental assistant; DS: dental surgeon; HIV: human immunodeficiency virus; SAS: specialized attention service.

* Chi-square test.

## DISCUSSION

Our results indicated that most of the interviewed professionals were White and female, and 59.1% of DS and 50% of DA had less than 5 years of professional activity. Many studies have reported these sex and ethnicity characteristics as the most prevalent among oral health care professionals.^5^^,^^13^^,^^14^ Azodo et al.^9^ observed that 66.7% of the DS evaluated in their study had less than 5 years of professional activity. Among the participants of our study, 93.5% had the 40-hour weekly workload of the public health service. Additionally, 77% of the DS reported having another professional activity. Shaghaghian et al.^12^ observed that 24.1% of the interviewed DS worked in the public health service and, of these, 9.7% also had another professional activity.

Regarding PPEs, the gloves were the most frequently mentioned (used by 100% of the interviewed professionals). In a study performed in Iran with dentistry students and professors, all participants also reported wearing gloves during the procedures.^15^ However, a study considering DS in the municipality of Montes Claros, Brazil, showed that only 88.5% of professionals used gloves full-time.^16^ Milfont et al.^17^ reported that gloves were the only PPE that was constantly used by professionals, since they have a visible function of protecting against external factors. On the other hand, we identified that goggles were one of the least frequently used PPEs, as 50% of the respondents reported using them only sometimes or never. Our data are in agreement with other studies.^18^^,^^19^ Despite the knowledge about infectious diseases and labor practices, professionals did not seem to comply with protocols such as those regarding the use of PPE, thus increasing the risk of exposure to biological materials.^20^

Since dental procedures involve the presence of aerosols and saliva droplets that come into direct contact with the professional, mask use is crucial for preventing pathogen transmission. Bragança et al.^18^ identified a satisfactory number of professionals who used masks (95.2%). According to a study performed in Iran with DS and DA, 97.9% of the professionals used disposable masks that were changed between patients,^21^ a fact that contrasts with our result that 63% of professionals reported not changing masks between patients. The lack of frequent mask changes results in an increase in humidity, which renders the mask not as efficient as a protection barrier.^5^

During dental treatments, DS and DA work directly or indirectly with the patient and in activities with risk of exposure to blood and other biological materials; this represents a risk of occupational transmission of HIV, hepatitis B, hepatitis C, and tuberculosis.^2^^,^^22^ We found that although 34.8% of the interviewed professionals reported having treated patients with tuberculosis, 30.1% (14) were not aware of the necessary preventive measures in these cases. Orth et al.^23^ evaluated the knowledge of oral health care teams (DS and DA) of 14 FHS units in the city of São Carlos, state of São Paulo,^23^ on tuberculosis and reported that 100% of the interviewees acknowledged that during the dental treatment they should be able to identify suspected tuberculosis cases. However, only 66% implemented behaviors such as the use of PPE and actions aimed at prevention, minimizing the risk of cross-infection. Other studies have reported the need for periodic training on tuberculosis in multidisciplinary teams.^24^ These results highlight the importance of including information regarding the oral health care of patients with tuberculosis in the continued education of teams at FHS and PHC units.

Periodic examinations such as tuberculin, HIV, and anti-HBs tests are control measures present in the workers’ health-disease process and are among the actions aimed at health workers as a vulnerable population.^1^^,^^12^ According to a study with health professionals of the city of Rio Branco, state of Acre, 37.8% sought examinations periodically, but 62.2% only underwent tests when they became ill.^25^ In our study, 58.7% of the interviewees reported periodic testing for HIV, 45.7% (21) for hepatitis C, and 47.8% (22) for hepatitis B. The tuberculin test was performed by 45.7% of the interviewed professionals. The current literature reports some difficulty in establishing periodic tuberculin testing due to lack of adherence by health professionals, which hinders the identification of latent tuberculosis infections.^24^

Vaccination is essential in the prevention of diseases to which the oral health care team is exposed on a daily basis.^26^ Regarding the hepatitis B vaccine, our results reported that 1 (2.4%) professional was not vaccinated and 5 (10.9%) had incomplete vaccination schedules. Nevertheless, 86.4% of the DS and 87.5% of the DA had complete vaccination schedules. In the United Kingdom, a study reported that 97% of the DS were immunized against hepatitis B,^27^ whereas a study in Nigeria showed a low prevalence of hepatitis B vaccination (68.6%) among DS and DA.^28^ Better guidance and recommendations to practitioners should favor the development of strategies to increase vaccination coverage.

As for the hepatitis B immunization results, 89.4% of the DA and 56.3% of the DS were anti-HBs reactive (had an immune protection against hepatitis B). Vieira et al.^29^ conducted a study with 58 students of health-related undergraduate courses in the city of Santa Maria, state of Rio Grande do Sul, where the group that had a complete vaccination schedule developed anti-HBs antibodies in 82.1% of cases, while 72.7% of those with incomplete vaccination had antibodies and 12.5% had antibodies even though they were not vaccinated. In Saudi Arabia, a study observed high percentages of DS vaccinated against hepatitis B (80.5%), but only 48.6% presented high antibody levels.^13^ Researchers have reported that part of the vaccine’s protection against hepatitis B is derived from the established immunological memory; antibodies remain in the serum for at least 15 years after the complete (3-dose) vaccination schedule and decrease over time, being reactivated if necessary.^14^

The prevalence of needlestick injuries among DS in recent years in Brazil ranged from 26% to 75%.^19^ In a needlestick accident, the probability of acquiring hepatitis B is greater than that for HIV and may reach 40%. For hepatitis C, this risk ranges from 1% to 10%. Although hepatitis B is considered the occupational disease with the highest risk for the oral health care team, cases of hepatitis C have been increasing and have become a public health concern.^30^ In the present study, 39% of the professionals had experienced accidents at least once during their professional activities. The frequency of accidents with needlestick injuries in this study was higher among DS (59.1%) than DA (20.8%), which relates to the more frequent direct contact of DS with these instruments compared to DA.

PEP is a protocol offered by the Brazilian Ministry of Health aimed to standardize procedures and guide the professionals in case of a workplace accident in the health care environment.^26^ We observed that PEP procedures were known by 65.2% of DS and DA, but the frequency of adequate reports of postperforation procedures was only 65.2% (30). The results of this study disclose the inadequacy of educational programs for oral health care professionals and the necessity of emphasizing the PEP protocol. Alavian et al.^15^ reported the need to incorporate biosafety and PEP protocols among the subjects of dentistry undergraduate courses to better prepare professionals for their clinical routine.

No cases of occupational infectious diseases were found in the participants of this study. Garcia et al.^19^ reported that contamination by hepatitis B, hepatitis C, and HIV was rare in dentistry; however, one exposure episode is all that it takes for transmission to occur. The biological risk is the most frequent, though neglected risk factor during patient care, favoring the risk of crossinfection.^19^

## CONCLUSION

Currently, the Brazilian regulatory standard NR32 establishes basic guidelines for guaranteeing the safety and health of health service workers. This study evaluated the prevalence of infectious diseases among oral health care workers and no positive cases were identified. As limitations, this study had the partial adherence of professionals due to lack of time or interest in participating in the study, absences on interviews even with prior scheduling, or the fact that some workers did not agree to undergo blood collection, which decreased our number of participants. However, when assessing the prevention measures adopted by these professionals, we found that some pieces of PPE were not used or misused even though workers were aware of the risks to which they are exposed during practice. Moreover, we also observed some failures in compulsory hepatitis B vaccine and a considerable history of needlestick accidents.
